# The Effect of Social Relationships on the Well-Being and Happiness of Older Adults Living Alone or with Relatives

**DOI:** 10.3390/healthcare11020222

**Published:** 2023-01-11

**Authors:** Cristina Fernandez-Portero, Josué G. Amian, David Alarcón, María J. Arenilla Villalba, José A. Sánchez-Medina

**Affiliations:** Department of Social Anthropology, Psychology and Public Health, Universidad Pablo de Olavide de Sevilla, 41013 Sevilla, Spain

**Keywords:** social relations, well-being, happiness, lives with relatives, living alone, social relationships

## Abstract

The aim of this study is to analyze the effect of the type of habitation of older adults—with relatives or alone—on their health and well-being. The participants were 352 people over 65 years of age who collaborated with the research on a voluntary basis. The data indicated that those who live with family members have better social integration, well-being and happiness than those who live alone. A multiple regression analysis showed the positive effect of living with relatives on well-being and happiness. However, participation in social activities mitigates the effect of the type of cohabitation explaining better well-being and happiness in the older population. These findings support the idea of designing and implementing intervention policies focused on activities that promote social participation and social interactions to improve well-being and happiness in the older adults.

## 1. Introduction

In 2018, people over 65 years of age in Europe represented 19.7% of the total population, with a total of 101 million older adults. Prospective studies estimate that this figure will rise to 149 million by 2050. The European older adult population is characterized by a higher percentage of women compared to men (for every 65-year-old man, 1.32 women are identified) and women living alone (40.4% of women compared to 22.4% of men living alone) [1].

Among the European countries with the largest number of older adults, Spain ranks fourth with 9.1 million older adults, preceded by Germany (17.9 million), Italy (13.8) and France (13.5). The National Statistical Institute (INE)’s prospective studies (2018–2068) forecast that the number of older adults in Spain will rise to 14 million (out of a total of 48,531,614 million inhabitants) by 2068, representing 29.4% compared to 19.3% in 2019 (INE, 2018). The number of women in Spain is 32% higher (5,145,437) than men (3,911,756). In addition, 60.2% of the older adults are married (75.8% of men, 48.0% of women), and 28.2% are widowed (12.0% and 40.9%, respectively) (2018). Of the 4,849,900 million people living alone in Spain, 2,131,400 are over 65 years of age. Of these, 1,511,000 (70.9%) are women. This figure reflects the high incidence of older adults living alone in Spain, especially widowed older adult women. Living alone is related to the health, autonomy, and social relations of older adults and to their life satisfaction or well-being and happiness.

Given the increasing number of older adults living alone, research has focused on analyzing the consequences of loneliness on health problems and cognitive decline associated with aging [2,3]. Most studies have shown how social isolation reduces the autonomy of the older adults while increasing age-associated chronic diseases [4]. According to Tamminen et al. [5], research with older people has mainly focused on the relationship between living alone and some elder pathologies, such as cognitive decline, health problems, depression, social isolation, etc. However, Tamminen et al. [5] suggest the need to investigate the association between living alone and some positive psychological dimensions of mental health, such as life satisfaction, well-being, and happiness, in order to analyze the protective factors of these dimensions. Specifically, the systematic review by Tamminen et al. [5] proposes that, among the population living alone, positive mental health is associated with quality of life and social relationships. Following the recommendations of these authors, the main objective of the current study is to analyze the relationship between the type of cohabitation and the well-being and happiness of older adults, after controlling for sociodemographic variables.

Previous studies have shown that loneliness negatively affects the well-being of older adults [6,7,8]. According to Lim and Kua [6], a study of older adults showed that living alone is associated with worse well-being than those who live with others. In a longitudinal study, De Vaus et al. [7] found evidence that older adults living alone have worse well-being than those living with relatives. It appears that living with others is related to the maintenance of well-being and happiness in the aging process. In turn, well-being and happiness are significantly reduced in older people who move to live alone, either because of changes in family structure or the loss of partners and friends [8]. However, other factors related to living with relatives and that affect the well-being and happiness of older adults, such as self-perception of health, have been studied [9]. It has been shown that self-perception of health is related to the type of cohabitation: older people who live alone are at greater risk of suffering health problems. However, Pasanen et al. [10], in a study on the subjective health of people living alone, pointed out that the relationship between living alone and subjective health is very heterogeneous depending on the age range. Thus, older people living alone have worse subjective health than young adults. In the case of young adults, living alone may be an indicator of independence and quality of life, while older people living alone have a lower quality of life and have to deal more with health problems. In addition, numerous studies have shown that self-perceived health has positive effects on the well-being and happiness of older adults [11]. Health problems associated with living alone can reduce the perception of well-being. Thus, San Román et al. [12], in a study on well-being in older adults, found that living with relatives is associated with a high self-perception of health and correlates with high levels of well-being or happiness in older adults, independent of other characteristics such as income or level of education.

Self-perception of health status is further related to the maintenance of autonomy—activities of daily living—that allow the older adults to maintain their independence from previous stages [13]. In turn, autonomy is favored by the type of coexistence—living alone or accompanied [14]. A study with older adults showed that living alone is associated, among other variables, with greater difficulty in performing activities of daily living and with poorer health and well-being. The importance of activity is defended by authors such as Parra-Rizo [15], who, in a study with active older adults, found evidence that being active and maintaining autonomy favors well-being in older adults.

Living with relatives, in addition to being associated with older adults’ health status, daily activities and functional autonomy, is directly related to a greater frequency of social interactions and a better support network. In turn, the social interactions of older adults, including contact with friends or relatives, constitute one of the main predictors of well-being [16,17]. In a study with older people without cognitive impairment, Etxeberria [18] found that the role of social relationships and autonomy in the aging process as promoters of well-being or life satisfaction is evident, and within social relationships, the family stands out as the main provider of social support. In this sense, the study of Rogers and Mitzner [19] showed the usefulness of technology as an opportunity to improve social connections with family and friends, which results in improved autonomy, health and well-being in older adults.

In the family environment, close and affectionate relationships are formed, and secure social support is established [20]. Thus, family relationships have a direct relationship with the well-being and health of older people and become more important with the passing of the years and the possible decline of social support networks [20,21]. In a recent study with subjects aged 16 to 85 years, those who had more family and social relationships had a lower incidence of depression and better health, especially among older adults [22]. In addition, older adults who have good relationships with their family members have better health and greater happiness and well-being throughout life [23]. However, older adults who do not live with their relatives have worse well-being [17]. It is evident that those who live accompanied or with relatives have better well-being indices than those who live alone.

Several studies with older adults have shown that leisure activities—physical, cultural, and outdoor activities—contribute directly to the level of well-being, which in turn improves mental and physical health [24,25,26,27]. Along these lines, those who live with family members tend to participate more in leisure activities and have free time [28,29,30,31]. In a recent study with older adults, it was found that even older people who have a relatively strong network of family and friends are more likely to engage in leisure activities [32]. Participation in leisure activities by older adults is associated with improved well- being [33,34].

Similarly, the quality of the environment of older adults is associated with greater well-being and health, especially among older adults who live alone [35]. However, living with relatives is associated with a better environment close to the subject—natural, human and physical—and, as a whole, predicts greater well-being and happiness [36,37]. In a study with older adults, Al Bahar et al. [38] found a direct relationship between environmental quality and getting along well with family and friends. Likewise, the quality of the environment is related to well-being, as evidenced by the study of Christina Hart et al. [39] with a European population, who found that living in neighborhoods in good condition, with more water and green spaces, is associated with being happier, and contact with natural spaces has positive effects on the health and well-being of older adults [40].

The aim of the present study is to analyze the effect of the type of cohabitation of older adults—with relatives or alone—on their positive mental health, well-being and happiness. However, according to Tamminen et al. [5], the current study examines other factors that may be associated with the well-being and happiness of older people living alone, such as health, quality of life, and social relationships. In line with previous studies, we hypothesize that the cohabitation of older adults with their relatives is a predictor of health, autonomy, and social support that, together, are directly associated with greater well-being and happiness. In contrast, older people living alone have poorer perceived health, autonomy, social support networks and leisure activities, all of which are associated with lower levels of well-being and happiness.

## 2. Materials and Methods

### 2.1. Participants

The selected sample consisted of 352 persons over 65 years of age who collaborated with the study on a voluntary basis. The distribution of the sample was homogeneous with respect to sex (50.6% women and 49.4% men). A total of 46.6% had no education compared to 34.9% with primary education and 19% with middle or higher education. With regard to age, 25.6% were between 65 and 69 years old, 24.4% were between 70 and 74 years old, 31% were between 75 and 79 years old and 19% were over 80 years old. In addition, 46.2% lived alone or without family members, and the remaining 53.8% lived with family members. In terms of the level of monthly income, 67.6% had an income of less than EUR 900, and 32.4% received an income of more than EUR 900 per month (see Table 1).

### 2.2. Instruments

The Brief Quality of Life Questionnaire (CUBRECAVI) [41] is a standardized instrument that measures the quality of life through 9 dimensions: health (subjective, objective and psychological); social integration (satisfaction with living together, frequency of social relations and satisfaction with social relations); functional abilities (functional autonomy and activities of daily living); activity and leisure (level of activity, frequency of activities and satisfaction with activities); environmental quality (satisfaction with environmental elements and general satisfaction with housing); satisfaction with life; income; and social and health services and education. It also includes a subjective measure of perceived quality of life measured in a single item. Based on the multidimensional concept of quality of life and health proposed by the WHO, this questionnaire allows a quick exploration of the most relevant components of the quality of life of older adults, both objective and subjective. The response options respond to a Likert-type scale from 1 to 4; the more positive the item is, the more likely it is to be 4. The reliability of the health scale was α = 0.839, that of social integration was α = 0.648, that of activity level was α = 0.723, that of functional abilities was α = 0.861 and that of environmental quality was α = 0.510.

The satisfaction with life scale (SWLS) [42] measures the well-being of people in their lives. It is a Likert scale with seven Likert-type response options ranging from 1 (Strongly disagree) to 7 (Strongly agree). Higher scores indicate higher well-being. The reliability of the scale was α = 0.812.

The Oxford Happiness Questionnaire (OHQ) [43] measures happiness through 29 Likert-type items, with response options ranging from 1 (strongly disagree) to 6 (strongly agree). The higher the score, the greater the happiness. The reliability of the scale is high (α = 0.804).

### 2.3. Procedure

Data collection was carried out in senior centers in Seville. The data were collected over 6 weeks. The participants were selected intentionally through agreements signed with the senior centers. Prior appointments were made with those responsible for the institutions and with the participants to inform them about the study and to collect signed informed consent. The study was approved by the University Ethics Committee (number 22/3-3) and met with the requirements of the Declaration of Helsinki. The questionnaires were administered by two researchers trained in the application of the scales to older adults.

The tests were implemented in small groups of 8 participants to ensure the older adult participants’ understanding of the test instructions, as well as the correct completion of the instruments. The sessions did not exceed 40 min, including rest times between tests to avoid fatigue effects.

### 2.4. Data Analysis

Statistical analyses were performed using SPSS version 23, employing a statistical significance at α = 0.05. Descriptive analyses were used to describe the sample characteristics (i.e., sociodemographic). A multivariate analysis of variance (MANOVA) test was used to assess differences by sex and type of cohabitation, controlling for age as a covariable. Pearson correlation analyses were performed to evaluate scale measure associations. Hierarchical multiple regression analysis was computed to examine associations for variables predicting well-being and happiness. Multicollinearity was tested by using the variance inflation factor (VIF), which measures the strength of correlation between the predictor variables in a regression model. In the reported regression equations, the VIF of the predictor variables was close to 1 and, did not exceed 2, indicating a low multicollinearity between the variables.

### 2.5. Ethical Considerations

All the procedures of this study were approved by the University Pablo de Olavide Research Ethics Commission with Human Beings (CEIH) (number 22/3-3) and followed the indicators of the International Conference of Good Clinical Practice by the 1975 Declaration of Helsinki guidelines.

## 3. Results

To study the relationship between the health, well-being and happiness of people over 65 years of age, the means of each of the variables under study were analyzed comparatively according to the type of cohabitation—alone or accompanied—and sex. The data analysis shows significant differences by sex and type of cohabitation (see Table 2).

In general, we found statistically significant differences by type of cohabitation; those living alone had a higher age range (χ^2^ (3, 351) = 17.88, *p* = 0.00), lower educational level (χ^2^ (2, 351) = 7.54, *p* = 0.02), and lower average income level (χ^2^ (1, 351) = 20.11, *p* = 0.00). In contrast, in a multivariate analysis of variance (MANOVA) test controlling for age in years as a covariable, people living with their relatives had a higher degree of social integration (*F*(1, 345) = 29.05, *p* = 0.000), more functional skills (*F*(1, 345) = 5.08, *p* = 0.025), greater well-being or life satisfaction (*F*(1, 345) = 7.73, *p* = 0.006) and greater happiness (*F*(1, 345) = 9.18; *p* = 0.003). The interaction effect between sex and type of cohabitation was significant in the health measure (*F*(1, 343) = 4.65, *p* = 0.032) and activity level (*F*(1, 346) = 5.14, *p* = 0.024). In relation to health, in general, women have better health (3.26 ± 0.38) than men (3.13 ± 0.41), but women who live in families (3.32 ± 0.35) have better health than those who live alone (3.17 ± 0.41). However, men who live alone have better health (3.15 ± 0.41) than those who live in a family (3.11 ± 0.40). In terms of the level of activity, women had higher levels of activity (2.44 ± 0.94) than men (2.10 ± 0.84), especially those who lived with a family (2.62 ± 0.97) compared to those who lived alone (2.19 ± 0.84). Regardless of gender, those who live with a family engage in more leisure activities, have more autonomy and functional abilities and have a better environmental quality than those who live alone. In the rest of the variables studied, leisure, functional skills, and environmental quality, there were no statistically significant differences. Gender differences were controlled by introducing gender as a predictor variable in the regression analyses.

Next, we performed a Pearson correlation analysis between age, sex, health, environmental quality, social integration, activity level, functional skills and leisure with well-being and happiness in Table 3, including the partial correlation coefficients controlling for sex. Controlling for the effect of sex, age was negatively associated with leisure activities (*r* = −0.313, *p* = 0.000), social integration (*r* = −0.128, *p* = 0.017), health (*r* = −0.194, *p* = 0.000), functional skills (*r* = −0.152, *p* = 0.004), and activity level (*r* = −0.236, *p* = 0.000). Health was positively associated with functional skills (*r* = 0.353, *p* = 0.000), leisure activities (*r* = 0.323, *p* = 0.000), social integration (*r* = 0.184, *p* = 0.001) and with activity level (*r* = 0.274, *p* = 0.000). Leisure activities correlate positively with functional abilities (*r* = 0.494, *p* = 0.000), social integration (*r =* 0.390, *p* = 0.000) and activity level (*r* = 0.374, *p* = 0.000). Well-being correlates positively with the rest of the variables except for age. In the case of happiness, there is a negative correlation with age (*r* = −0.221, *p* = 0.000) and a positive correlation with the rest of the variables. In addition, the data indicated a positive correlation between well-being and happiness (*r* = 0.493, *p* = 0.000).

The aim of this study was to demonstrate that the health, social integration, activity level, functional skills and leisure activities of older adults influence their well-being and happiness. In this sense, a two-step multiple regression analysis was performed, taking well-being and happiness as dependent variables and age, sex, income level and the person with whom they live as predictor variables in Model 1, adding the rest of the psychosocial variables (health, social integration, activity level, leisure, functional skills and environmental quality) as predictors in step two of the model (Table 4).

Analyzing the contribution of each of the variables introduced as predictor variables of well-being, it was observed that the variable in the first step of the model that makes the greatest contribution is the type of cohabitation (β = 0.138, t = 2.435, *p* = 0.015). When we introduce the rest of the psychosocial variables, those that contribute most to the model are age (β = 0.153, t= 2.793, *p* = 0.006), health (β =0.191, t = 3.395, *p* = 0.001), social integration (β = 0.120, t = 2.083, *p* = 0.038), leisure activities (β = 0.143, t = 2.159, *p* = 0.032) and environmental quality (β = 0.137, t = 2.712, *p* = 0.007).

The variables introduced as predictor variables of happiness indicate that the variables that contribute significantly in Model 1 are age (β = −0.181, t = −3.352, *p* = 0.001) and cohabitation (β = 0.170, t = 3.085 *p* = 0.002). In the second step of the model, once the psychosocial variables are introduced, the variables that contribute the most to the model are health (β = 0.137, t = 2.568, *p* = 0.011), social integration (β = 0.260, t = 4.790, *p* = 0.000), and activity level (β = 0.216, t = 4.056, *p* = 0.000).

## 4. Discussion

The progressive increase in older people living alone has led to a growing number of studies on the aging process, focused mainly on the analysis of health indicators and cognitive functioning [2,3]. The aim of this study is to analyze the association of the type of cohabitation of older adults with their well-being and happiness, in addition to understanding the relationships with other psychosocial variables such as autonomy (functional skills) and social relationships (social integration, level of activity, leisure, and environmental quality). Our hypothesis was that the cohabitation of older people with their relatives is associated with a higher degree of perceived health, autonomy and social support, which together are related to greater well-being and happiness. In contrast, older people living alone have poorer perceived health, autonomy, social support networks, and leisure activities, all of which are associated with lower well-being and happiness.

Older people in our study living at home with relatives had higher levels of life satisfaction and happiness. These data are consistent with previous studies that found higher levels of well-being associated with living with others in the aging process [6,7,8]. Similar to previous studies, the sociodemographic profile of older people living alone was that of a person of high average age, lower educational level, and lower income level than people living with relatives [44]. This sociodemographic profile of older people living alone reflects economic and cultural disadvantages that, in turn, enhance health problems and reduce their quality of life. In addition, older people living alone had lower rates of functional skills [15] and less social integration [18,21]. Thus, we found that older people living alone have risk factors for loss of autonomy and social support, which, in turn, impair their health, well-being and happiness [17]. This evidence may have socio-emotional implications for older people living alone [45,46]. According to Bericat [45], it has been claimed that emotions are induced by interpersonal situations, and given the social nature of emotions, social interaction will affect our well-being and happiness.

Living alone is also related to suffering from greater health problems [9,10] and increases the risk of having poorer health and less independence or autonomy [6,7]. In contrast, living with relatives seems to strengthen social networks; the family members with whom the older adults coexist provide social relationships, enriching their social network [22,23]. Consequently, living with relatives is associated with greater autonomy and social integration, which, in turn, is associated with better health, well-being and happiness [47]. In summary, older people living alone, as previous studies have shown, are at greater risk of health problems, loss of functional skills and social relationships. Taken together, these factors are interrelated, and the lack of one will precipitate the difficulties of the others; thus, the loss of functional skills is an impediment to maintaining social relationships and reduces control over the management of their health problems.

The aging process requires studying relationships with family and the type of cohabitation—alone or accompanied—to explain health, well-being and happiness [21]. This study has shown that people who cohabit with their relatives have higher levels of satisfaction and happiness. These data support our hypotheses by finding significant differences in wellbeing and happiness between those who live with family and those who live alone. However, the main finding of our study is that well-being and happiness are explained by the social relationships and activity level of the older adult, regardless of the type of cohabitation. Controlling for the type of cohabitation, social support factors and the activity level of the older adults accounts significantly for the levels of well-being and happiness. This evidence is consistent with the study of Djundeva et al., [47] who found that the well-being of older adults depends on diverse networks and not on the type of cohabitation. These findings support the hypothesis that, even among those living alone, social activities and interaction would enhance the positive mental health of older people. According to Tamminen et al. [5], beyond the type of cohabitation, social relationships and activities have a positive effect on the well-being and happiness of older adults [21]. Thus, our data showed how having good social integration, participating in leisure activities, engaging in good levels of activity, and having a close and familiar environment favor well-being and happiness, controlling for the type of cohabitation. That is, living with relatives helps to be well and happy after 65 years of age, but those who live alone but maintain high levels of social integration, participation in social activities and support networks also are able to maintain a high level of health, well-being and happiness.

Thus, the perception of health is positively associated with the maintenance of functional abilities [13], which allow older adults to maintain independence and personal autonomy and are key to having a high level of activity, participating in leisure activities and maintaining a good social network, as well as having a good perception of the quality of the environment [39]. Our data showed a high correlation between all the variables of the study and evidenced the network of social relationships as an explanatory factor of well-being and happiness in line with previous studies [18]. We also found evidence of the importance of leisure activities when performed in social interaction on the well-being of older adults [32].

Among the limitations of our study is not having observations of the measures at different points in time, so the results cannot be interpreted causally, and we can only point out the association between variables. Likewise, the comparisons by age are cross-sectional, but we do not know how the indicators measured may change over time. An additional weakness of the study is that it does not experimentally compare older people who have been randomly assigned to a prescribed or nonprescribed social activity but rather records the frequency of activities they perform on a regular basis. Future research on aging should focus on experimentally analyzing the effect of the social participation of the older adults in their environment and community, prescribing on the improvement of their well-being and health, especially among those who live alone [35]. Other aspects to consider for future research are to analyze the socioemotional implications of the type of cohabitation [45,46], and the sociodemographic reality of older adults to help understand their lives. Although previous studies have shown that well-being and happiness are often associated with sociodemographic variables such as sex, education, and income, in our results no such associations were observed. This may be because the sample was composed only of people over 65 years of age drawn from the same population. Previous studies reveal that among older people the level of well-being and happiness increases with respect to previous stages of the life cycle [48], and in older people it is strongly associated with social functioning and social support [49]. For this purpose, it would be necessary in future research to compare the levels of well-being and happiness associated with sociodemographic and quality of life variables between adults and the elderly, as well as to draw a representative sample with a higher degree of heterogeneity of population characteristics. In addition, longitudinal studies are needed to obtain conclusive results on the causal relationship between the variables and the changes observed with age.

## 5. Conclusions

Previous studies show the influence of the type of cohabitation, living with relatives versus living alone, on the well-being and happiness of older people [5]. However, engaging in social activities that involve the relationships of the older adults with others in their environment improves the health, well-being, and happiness of the older adults and reduces the risks associated with living alone. These findings support public policies aimed at designing and implementing social intervention programs focused on social activities that promote participation and social interactions to improve the well-being and happiness of older adults, especially among people living alone. In this sense, investment in social participation policies based on lifelong learning has proven to be a good strategy for improving the well-being of the elderly [50,51]. Older people who engage in formal or informal learning activities have greater well-being. These learning activities are both a stimulus to keep their functional skills active and a means to interact socially and belong to groups where they can develop emotional ties and support networks.

## Figures and Tables

**Table 1:** Sociodemographic characteristics of the sample.

		*n* = 352	Lives Alone 162 (46.2%)	Live with the Family 189 (53.8%)
Sex	Man	178 (50.6%)	85 (52.5%)	88 (46.6%)
	Woman	174 (49.4%)	77 (47.5%)	101 (53.4%)
Age	From 65 to 69	90 (25.6%)	28 (17.3%)	62 (32.8%)
	From 70 to 74	86 (24.4%)	35 (21.6%)	51 (27.0%)
	From 75 to 79	96 (31.0%)	51 (31.5%)	45 (23.8%
	80 or more	79 (19.0%)	48 (29.6%)	31 (16.4%)
Education	No education	162 (46.0%)	66 (40.7%)	95 (50.3%)
	Primary	123 (34.9%)	69 (42.6%)	54 (28.6%)
	Medium or higher	67 (19.0%)	27 (16.7%)	40 (21.2%)
Income	Less than EUR 900	238 (67.6%)	129 (79.6%)	108 (57.1%)
	More than EUR 900	114 (32.4%)	33 (20.4%)	81 (42.9%)

Note: Column percentages for each variable are shown in parentheses. Row percentages in parentheses are used for type of cohabitation.

**Table 2:** Characteristics of the sample by sex and type of cohabitation, controlling for age in years.

*n* = 352	With Family Mean (SD)		Living Alone Mean (SD)		*p*
	Men (46.5%)	Women (53.5%)	Men (52.5%)	Women (47.5%)	
Age	73.53 ± 5.57	72.76 ± 6.06	76.31 ± 5.96	76.62 ± 6.56	0.000
Health	3.11 ± 0.40	3.32 ± 0.35	3.15 ± 0.41	3.17 ± 0.41	0.009
Social integration	2.80 ± 0.73	2.76 ± 0.71	2.28 ± 0.90	2.27 ± 0.83	0.000
Activity level	2.09 ± 0.85	2.62 ± 0.97	2.12 ± 0.83	2.19 ± 0.84	0.001
Leisure activities	2.14 ± 0.36	2.13 ± 0.34	2.01 ± 0.38	2.07 ± 0.40	0.389
Functional skills	3.32 ± 0.79	3.60 ± 0.62	3.22 ± 0.86	3.21 ± 0.87	0.025
Environmental Quality	2.80 ± 0.26	2.81 ± 0.25	2.87 ± 0.22	2.84 ± 0.22	0.431
Well-being	5.03 ± 1.15	5.25 ± 1.09	4.70 ± 1.56	4.84 ± 1.39	0.006
Happiness	4.16 ± 0.58	4.25 ± 0.55	3.89 ± 0.55	4.04 ± 0.53	0.003

Note. Statistically significant differences were found by sex in health, activity level and happiness, by type of cohabitation in age, functional skills, social integration, happiness and life satisfaction.

**Table 3:** Correlations between well-being and happiness with age and sex, health, social integration, activity level, leisure activities, functional skills and environmental quality.

	1	2	3	4	5	6	7	8	9
1.Age	1	−0.198 **	−0.128 *	−0.236 **	−0.314 **	−0.155 **	0.022	0.006	−0.224 **
2. Health	−0.194 **	1	0.182 **	0.294 **	0.324 **	0.362 **	0.084	0.266 **	0.274 **
3. Social support	−0.128 *	0.184 **	1	0.094	0.390 **	0.251 **	0.027	0.234 **	0.364 **
4. Level of activity	−0.233 **	0.274 **	0.096	1	0.373 **	0.316 **	0.051	0.172 **	0.324 **
5. Leisure activities	−0.313 **	0.323 **	0.390 **	0.374 **	1	0.495 **	0.083	0.245 **	0.301 **
6. Functional skills	−0.152 **	0.353 **	0.253 **	0.305 **	0.494 **	1	0.019	0.193 **	0.197 **
7. Environmental Quality	0.021	0.089	0.027	0.056	0.083	0.022	1	0.154 **	0.082
8. Well-being	0.009	0.258 **	0.235 **	0.161 **	0.243 **	0.187 **	0.156 **	1	0.497 **
9. Happiness	−0.221 **	0.262 **	0.366 **	0.311 **	0.300 **	0.189 **	0.085	0.493 **	1

Note. Partial correlations are in the lower part and the bivariate correlations are in the upper right part. * *p* < 0.05; ** *p* < 0.01.

**Table 4:** Hierarchical multiple regression analysis for variables predicting wellbeing and happiness.

Variable	Well-Being				Happiness			
	b	β	t	R^2^	b	β	t	R^2^
Model 1				0.035				0.087 ***
Age	0.067	0.063	1.139		−0.466	−0.181	−3.352 ***	
Sex	−0.860	−0.065	−1.228		−0.143	−0.098	−1.893	
Lives with relatives	1.815	0.138	2.435 **		5.447	0.170	3.085 ***	
Education	0.078	0.009	0.160		0.331	0.016	0.285	
Income	1.148	0.082	1.395		−1.156	−0.034	−0.593	
Model 2				0.171 ***				0.260 ***
Age	0.161	0.153	2.793 **		−0.221	−0.086	−1.663	
Sex	−0.509	−0.039	−0.760		−1.800	−0.056	−1.167	
Lives with relatives	1.273	0.097	1.732		2.689	0.084	1.590	
Education	−0.763	−0.088	−1.573		−1.183	−0.056	−1.059	
Income	1.154	0.082	1.488		−0.947	−0.028	−0.530	
Health	0.125	0.191	3.395 ***		0.218	0.137	2.568 **	
Social integration	0.190	0.120	2.083 *		1.007	0.260	4.790 ***	
Activity level	0.467	0.065	1.146 *		3.803	0.216	4.056 ***	
Leisure activities	0.228	0.143	2.159		0.269	0.069	1.107	
Functional skills	0.005	0.002	0.041		−0.220	−0.044	−0.776	
Environmental quality	0.623	0.137	2.712 **		0.752	0.068	1.424	
**Model Summary**	**Well-Being**				**Happiness**			
	**R**		**R^2^**	**F**	**R**		**R^2^**	**F**
Model 1	0.186 ^a^		0.035	2.473 *	0.295 ^b^		0.087	6.568 ***
Model 2	0.414 ^a^		0.171	6.359 ***	0.510 ^b^		0.260	10.845 ***

Notes. ^a^ Dependent variable: Well-Being. ^b^ Dependent variable: Happiness. * *p* < 0.05; ** *p* < 0.01; *** *p* < 0.001.

## Data Availability

The datasets used in the current study are available from the authors upon reasonable request and with permission of all the authors.
